# Succinate metabolism–related gene signatures enable diagnosis and prognostic stratification in early-stage hepatocellular carcinoma

**DOI:** 10.1515/med-2026-1465

**Published:** 2026-11-16

**Authors:** Xiaomei Wang, Yi Lu, Xiaoying Ye, Jiaojian Lv

**Affiliations:** Department of Infectious Diseases, The First Affiliated Hospital of Lishui University, Lishui People’s Hospital, Lishui, Zhejiang, China

**Keywords:** hepatocellular carcinoma, succinate metabolism, diagnostic model, prognostic signature

## Abstract

**Objectives:**

Succinate metabolism influences tumorigenesis through energetics, epigenetics, and immunity, yet the clinical value of succinate metabolism–related genes (SMRGs) in early-stage hepatocellular carcinoma (HCC) remains unclear.

**Methods:**

Using transcriptomic and clinical data from TCGA and GEO cohorts, we identified differentially expressed SMRGs (DESMRGs). Machine learning (LASSO, Random Forest, XGBoost) selected diagnostic biomarkers. Immune infiltration, immunotherapy response, and drug sensitivity were assessed via ssGSEA, CIBERSORT, TIDE, and pharmacogenomic databases.

**Results:**

Twenty-four DESMRGs were identified in early-stage HCC. CDKN2A, LIFR, NTF3, and PVALB showed strong diagnostic potential (each AUC >0.70). Consensus clustering based on SMRGs defined two subgroups with distinct survival and pathway activities. A six-gene risk score stratified patients into high- and low-risk groups, validated externally. High-risk patients exhibited an immunosuppressive microenvironment, potential differences in immunotherapy-related response, higher TIDE scores, and distinct mutation and drug sensitivity profiles.

**Conclusions:**

SMRGs may serve as potential diagnostic and prognostic biomarkers for early-stage HCC and are closely linked to immune phenotypes and treatment implications. These findings suggest a potential association between succinate metabolism-related patterns and tumor immunity, supporting SMRG-based models for clinical risk stratification and therapy decisions in HCC, pending further validation.

## Highlights

–Integrated differential expression analysis and WGCNA to identify 24 DESMRGs in early-stage HCC, and established a four-gene diagnostic signature with excellent performance in TCGA and external validation.
–Defined two succinate metabolism–related molecular subtypes with distinct overall survival and developed a subtype-derived six-gene prognostic model that achieved stable time-dependent accuracy in TCGA and GSE76427, and retained prognostic value after adjustment for available clinical variables.
–Linked the prognostic risk score to immune-related and computationally inferred therapeutic heterogeneity, characterized by broadly higher immune checkpoint expression, higher TIDE scores and potential differences in anti–PD-L1 response patterns in the high-risk group, alongside increased TMB and distinct predicted drug sensitivities with CellMiner-supported gene–drug correlations.

## Introduction

Hepatocellular carcinoma (HCC) is a leading cause of cancer mortality worldwide, responsible for approximately 8–10 % of cancer-related deaths [1], [2], [3]. Despite improvements in surveillance of at-risk populations (e.g. cirrhotic patients), early-stage HCC often remains underdiagnosed – fewer than 30 % of cases in Western cohorts are detected at very early or early stages (Barcelona Clinic Liver Cancer stage 0/A) when curative treatments are feasible [4]. Patients diagnosed at early stage and treated with resection, ablation, or transplantation can achieve 5-year survival rates of 50–70 %, approaching 70 % in ideal scenarios [5]. In contrast, those diagnosed at more advanced stages and managed with only palliative therapies face a median survival of roughly 1–2 years [6]. This stark survival gap highlights the critical importance of early diagnosis and accurate prognostic stratification in HCC [7]. However, achieving early detection is challenging. The current standard surveillance tools – ultrasound imaging with or without serum alpha-fetoprotein (AFP) – have suboptimal sensitivity for early HCC. Ultrasound-based surveillance misses up to one-third of early tumors due to masking by underlying cirrhosis and operator dependence [8]. Likewise, AFP alone is inadequately sensitive: only ∼10–20 % of very early HCC cases present with elevated AFP levels [9], 10]. Even combining ultrasound with AFP increases detection by only a small margin [11]. Thus, there is an urgent unmet need for novel biomarkers or molecular signatures that can complement imaging and improve early HCC diagnosis [12].

Growing evidence has positioned metabolic reprogramming as a key direction for identifying clinically useful biomarkers and refining molecular stratification in HCC [13]. Metabolic reprogramming is a hallmark of cancer, and HCC cells exhibit profound shifts in pathways of glucose, lipid, and amino acid metabolism to support growth in the nutrient- and oxygen-poor liver microenvironment [14]. Succinate, a dicarboxylic acid intermediate of the tricarboxylic acid (TCA) cycle, has gained particular attention as an “oncometabolite” that can drive tumorigenesis when abnormally accumulated [15], 16]. Succinate normally is oxidized to fumarate by succinate dehydrogenase (SDH), a mitochondrial enzyme complex (complex II of the electron transport chain) [17]. In certain tumors, SDH loss-of-function leads to pathologic succinate accumulation, which in turn inhibits α-ketoglutarate–dependent dioxygenases, causing epigenetic alterations and pseudohypoxia via stabilization of hypoxia-inducible factor 1α (HIF-1α) [18], 19]. Succinate can also be released into the tumor microenvironment, where it signals through its receptor SUCNR1 (GPR91) on immune cells and other stromal cells [20], [21], [22]. Through these non-canonical pathways, succinate has been implicated in cancer progression and immune evasion: for example, succinate accumulation can promote angiogenesis, induce HIF-1α–driven gene expression (including VEGF and PD-L1), and skew immune cells toward a protumoral, inflammatory phenotype [23], [24], [25]. Indeed, studies in immune cells have shown succinate acts as a signaling metabolite that drives macrophages to a pro-inflammatory, tumor-promoting state and impairs cytotoxic T-cell activity [26], 27]. These discoveries position succinate metabolism at the intersection of tumor cell-intrinsic metabolism and the immunosuppressive tumor microenvironment, making it a compelling focus for biomarker development.

In this study, we address the unmet need for robust biomarkers to improve the diagnosis and prognostic stratification of early-stage HCC, given the emerging but still insufficient understanding of succinate metabolism–related genes (SMRGs) in tumor biology. We hypothesize that SMRGs can distinguish early-stage HCC from benign liver tissue and are associated with patient prognosis as well as tumor immune microenvironment characteristics. Accordingly, the key research questions are: (1) which SMRGs can reliably discriminate early-stage HCC from non-malignant liver tissue based on transcriptomic data, and (2) whether a SMRGs-based risk score model can be constructed to predict overall survival in patients with early-stage HCC. Through this study, we hope to fill the knowledge gap linking cancer metabolism and clinical biomarkers in HCC, and to provide a foundation for new metabolism-based diagnostic and prognostic tools with potential translational significance.

## methods

### Data acquisition

RNA-seq expression profiles for HCC were obtained from the TCGA-LIHC cohort via the UCSC Xena platform (https://xena.ucsc.edu/). To restrict analyses to early-stage disease, only stage I–II samples were retained, comprising 260 tumor tissues and 50 non-tumor liver tissues for model development. Copy number variation (CNV) data for TCGA-LIHC, along with linked clinical annotations, were retrieved from the Genomic Data Commons (GDC) portal (https://portal.gdc.cancer.gov/). Samples without adequate survival follow-up were omitted prior to downstream survival analyses. External validation was performed using two independent Gene Expression Omnibus (GEO) microarray datasets (https://www.ncbi.nlm.nih.gov/geo/; GSE14520: non-tumor, n=220; tumor, n=225; and GSE76427: tumor, n=117). All expression datasets were log2-transformed as provided. To reduce within-cohort technical variability, normalization was performed independently for each dataset using the normalizeBetweenArrays function in the limma R package. No cross-platform merging or batch correction was applied, and each cohort was analyzed separately. For external validation, models were applied using the same gene set and coefficients, while risk groups were defined based on cohort-specific median cutoffs. In parallel, a curated list of succinate metabolism–related genes (SMRGs) was compiled from the GeneCards database (https://www.genecards.org/).

### Differential expression analysis and identification of DESMRGs

Differential transcriptional changes between tumor and adjacent non-tumor liver tissues were quantified with the limma framework. Genes meeting both an adjusted p value <0.05 and |log_2_FC| >1 were retained as differentially expressed genes (DEGs).

To delineate tumor-associated co-expression patterns, we constructed a weighted gene co-expression network based on the DEGs using weighted gene co-expression network analysis (WGCNA). An appropriate soft-thresholding power was selected to approximate scale-free topology, and co-expression modules were subsequently detected using hierarchical clustering with dynamic tree cutting. Candidate succinate metabolism–related genes were then refined by intersecting the DEGs list with the SMRG catalogue and key module genes, yielding the differentially expressed SMRG set (DESMRGs). To annotate the biological relevance of the key genes and DESMRGs, Gene Ontology (GO) and Kyoto Encyclopedia of Genes and Genomes (KEGG) pathway enrichment analyses were carried out with the clusterProfiler R package.

### Machine learning–based identification of key features

Machine-learning approaches were applied to further prioritize important variables from the DESMRGs. Specifically, Least absolute shrinkage and selection operator (LASSO) regression, Random Forest (RF), and XGBoost analyses were implemented in R using the glmnet, randomForest, and xgboost packages, respectively, to identify candidate feature genes [28], 29]. For LASSO regression, 10-fold cross-validation was performed and the optimal penalty parameter was determined using the lambda min criterion. RF analysis was conducted with ntree=1,000, and XGBoost analysis was performed using an eta parameter of 1. These machine-learning methods were selected because of their complementary strengths in high-dimensional feature selection and classification analysis. The overlap among genes selected by these three methods was then taken, and the intersecting genes were considered potential candidates with diagnostic value.

### Construction and evaluation of the diagnostic model

In the training cohort, discriminatory performance of the machine-learning–selected candidates was quantified by receiver operating characteristic (ROC) analysis implemented in the pROC package, with AUC values calculated for each gene. Genes with an AUC above the predefined cutoff (AUC>0.70) were retained as candidate diagnostic markers and were integrated to build the diagnostic model. A nomogram was constructed with the rms R package, and model calibration was evaluated by comparing predicted vs. observed probabilities using calibration plots. The model’s clinical net benefit was evaluated using decision curve analysis (DCA) across varying decision thresholds. The final model was then tested in an independent dataset (GSE14520) to verify its generalizability.

### Expression analysis and single-gene enrichment analysis of diagnostic markers

We compared the expression of the diagnostic genes between tumor and non-tumor liver tissues. For functional interpretation at the single-gene level, the GO biological process gene set (c5. go. bp. v2024. 1. Hs.symbols.gmt) was obtained from MSigDB (https://www.gsea-msigdb.org/gsea/msigdb). Each diagnostic marker was individually interrogated by GSEA using the clusterProfiler R workflow.

### Immune-related analyses

We used single-sample gene set enrichment analysis (ssGSEA) to calculate immune infiltration scores and immune function scores in HCC samples. These metrics were subsequently compared between tumor tissues and non-tumor controls. In parallel, cell-type deconvolution was performed with CIBERSORT, and MCP-counter scores were computed via the IOBR R package to provide complementary infiltration estimates. For each diagnostic gene, we computed Pearson’s r between its expression and the abundance of each immune-cell type. We further profiled immune signatures in the high- and low-risk groups using ssGSEA and CIBERSORT, and summarized the expression patterns of immune checkpoint genes across risk strata. We correlated CIBERSORT-inferred immune-cell abundances with model feature-gene expression using Pearson’s r. Finally, Tumor Immune Dysfunction and Exclusion (TIDE; http://tide.dfci.harvard.edu/) was employed to infer immune evasion and predicted response to immunotherapy, and TIDE scores were compared between risk groups.

### Immune therapy response prediction

Immune checkpoint blockade targeting PD-1/PD-L1 has become an important component of cancer immunotherapy. The IMvigor210 anti–PD-L1 cohort was used as an independent immunotherapy benchmark. Expression matrices and corresponding clinical variables were obtained through the IMvigor210CoreBiologies R package, and the risk score was evaluated for its ability to predict treatment response.

### Prognosis-related gene analysis

We matched gene-expression data to clinical follow-up information and removed cases with OS ≤30 days prior to model construction. Each DESMRG was subsequently tested by univariable Cox regression (survival package), and genes meeting p<0.05 were retained as prognostic candidates. For genes passing this filter, expression patterns were contrasted between tumor and non-tumor samples. A correlation matrix was generated across the prognosis-related gene set. The matrix was then presented as a heatmap to facilitate visualization of gene–gene associations. In addition, the genomic distribution of these genes was mapped to chromosomal coordinates and displayed with the RCircos package.

### Succinate metabolism–related subtype identification

HCC samples were grouped into SMRG-related subtypes by applying ConsensusClusterPlus to the expression matrix of the prognostic genes retained from univariable Cox screening (p<0.05). We visualized the clustering pattern in a reduced-dimensional space using principal component analysis (PCA) and assessed prognostic divergence among subtypes by Kaplan–Meier curves with log-rank tests (survival package). For transcriptomic characterization, limma was used to identify DEGs between subtypes (|log_2_FC| >1; FDR-adjusted p<0.05), followed by GO and KEGG enrichment with clusterProfiler.

### Development of a subtype-related prognostic signature

After matching gene-expression matrices to clinical outcomes, cases with very short follow-up (<30 days) were removed. We next screened subtype-derived DEGs for survival relevance using single-gene Cox models and retained those meeting p<0.05. Feature selection was performed with an L1-regularized Cox model (glmnet), where λ was chosen by cross-validation to balance fit and sparsity. A multivariable Cox model was subsequently fitted on the selected genes, and a linear predictor was used to compute patient-level risk scores. Samples were grouped into two risk categories based on the score, and survival separation was assessed with Kaplan–Meier analysis. Time-dependent ROC analysis (timeROC) was used to evaluate accuracy at 1, 3, and 5 years, and we visualized risk scores alongside survival outcomes to demonstrate stratification. The prognostic performance of the model was further examined in an independent validation cohort using the same evaluation framework (KM curves, time-dependent ROC, and risk distribution/survival status plots). In addition, each model gene was evaluated individually by Kaplan–Meier analysis in the training cohort, and expression patterns of signature genes were displayed with boxplots for tumor vs. control samples as well as across risk strata.

### Enrichment analysis

We assessed risk-related pathway activity using the GSEA desktop tool (v4.3.2), with ranked gene lists constructed from the high-vs. low-risk comparison. Differential expression between risk groups was then performed, retaining genes with |logFC| >1 and FDR <0.05. Functional interpretation of the resulting gene set was carried out by GO and KEGG over-representation analyses implemented in clusterProfiler.

### Independent prognostic analysis

To determine whether the model-derived risk score provides prognostic information beyond routine clinicopathologic variables, we fitted univariate and multivariable Cox models incorporating the risk score together with clinical covariates. Nomogram construction, calibration curves, and DCA were conducted as described in Section Construction and evaluation of the diagnostic model.

### Subgroup analysis based on clinicopathological features

We examined whether the risk score varied across key clinicopathologic categories by comparing risk-score distributions among clinical subgroups. Patients were then jointly classified by risk status (high vs. low) and clinicopathologic strata to assess the robustness of prognostic separation under different clinical contexts. Patients were partitioned into clinical subgroups based on age (≤65/>65), sex, stage, and T category. Survival separation between risk groups was examined in each subset using Kaplan–Meier curves.

### Tumor mutational burden analysis

We estimated tumor mutational burden (TMB) on a per-sample basis using TCGA-LIHC somatic mutation data. The resulting TMB values were compared between risk groups and visualized as violin plots. In parallel, the mutation landscape of the 20 most frequently altered genes was summarized, and an oncoplot (waterfall plot) was generated using the maftools R package.

### Drug sensitivity prediction

Drug–gene associations were queried using the CellMiner resource (https://discover.nci.nih.gov/cellminer/) to prioritize compounds whose activity profiles showed significant relationships with the prognostic signature genes. We used pRRophetic to estimate the half-maximal inhibitory concentration (IC50) values from gene-expression profiles for each patient. The predicted IC50 distributions were compared between risk groups, and lower values were taken to reflect increased drug sensitivity.

### Statistical analysis

All computations were carried out in R (v4.4.0). Two-group comparisons were performed using the Wilcoxon rank-sum test. Time-dependent ROC curves were generated in R with the timeROC package. Corresponding AUCs were used to summarize discriminatory ability. Figures were generated primarily with ggplot2. Unless otherwise specified, statistical significance was set at p<0.05, and p values were denoted by the following asterisk code: ****, p<0.0001; ***, 0.0001≤ p<0.001; **, 0.001≤ p<0.01; *, 0.01≤ p<0.05; ns, p≥0.05.

## Results

### Identification of DEGs and key WGCNA module in HCC

“Differential transcriptomic profiling revealed 3,503 genes with significant expression changes between tumor and non-tumor liver tissues, comprising 1,592 upregulated and 1,911 downregulated genes (Figure 1A). We built a WGCNA network from the DEGs. After evaluating scale-free fit across candidate powers, we selected β=5 to transform the correlation matrix into an adjacency matrix (Figure 1B and C). Six modules were detected, and their eigengenes were correlated with tumor status, with the turquoise module showing the strongest signal (Figure 1D). Within the turquoise module, 237 genes with high module membership and gene significance (MM >0.7 and GS >0.4) were prioritized as key genes (Figure 1E). Functional enrichment of the key genes consistently pointed to cell-cycle control and mitotic progression. In GO terms, the top signals clustered around chromosome dynamics during division, including sister chromatid segregation, chromosome segregation, and mitotic nuclear division (Figure 1F). KEGG analysis further supported this theme, highlighting pathways such as cell cycle, oocyte meiosis, and cellular senescence (Figure 1G). To focus on succinate metabolism–related dysregulation, DEGs, key genes, and SMRGs were intersected and yielded 24 DESMRGs (Figure 1H). Enrichment analysis of these 24 DESMRGs further supported their close association with cell-cycle phase transitions and mitotic regulation, and KEGG results similarly implicated cell cycle– and cancer-related pathways (Figure 1I).

**Figure 1:**
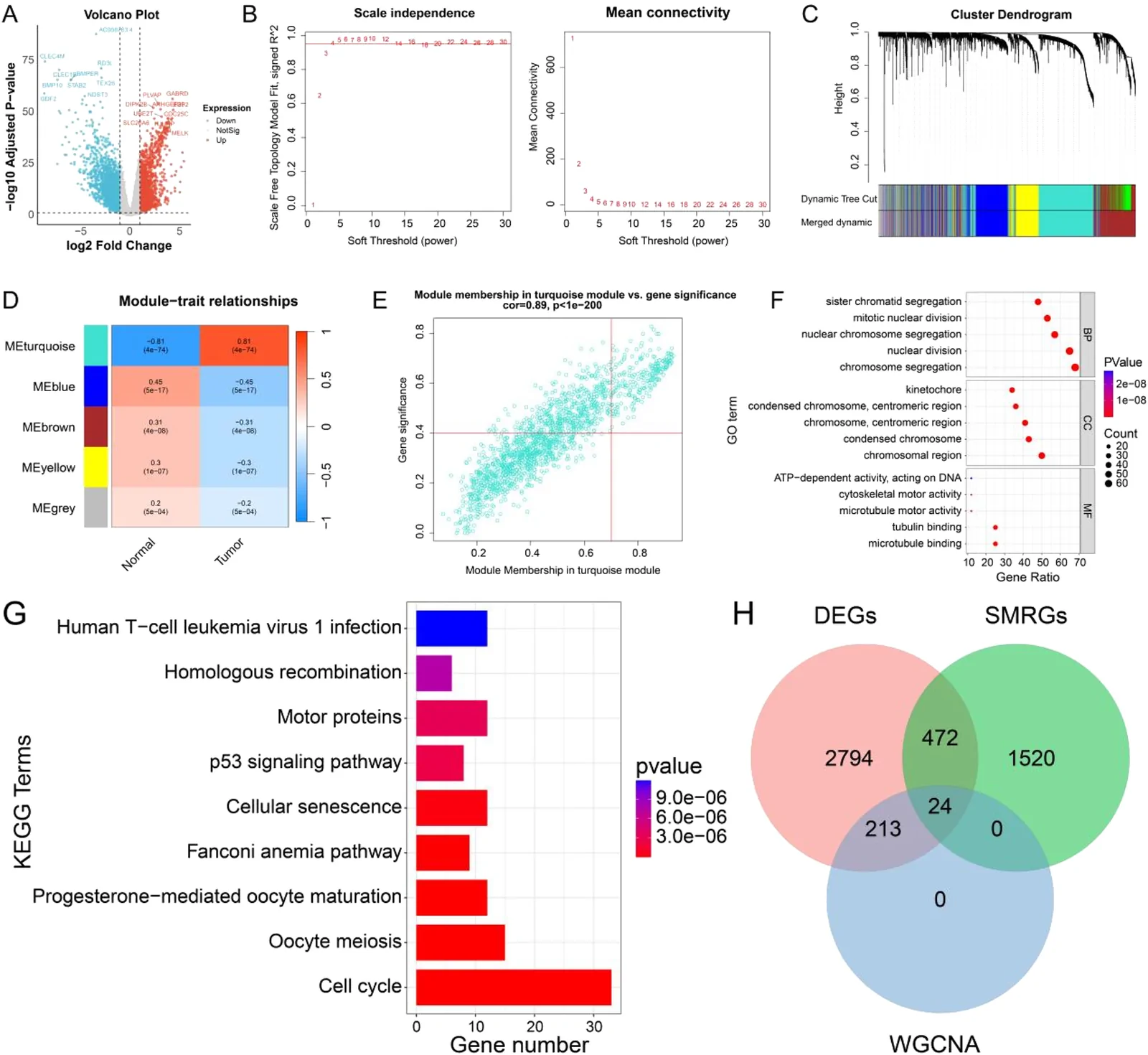

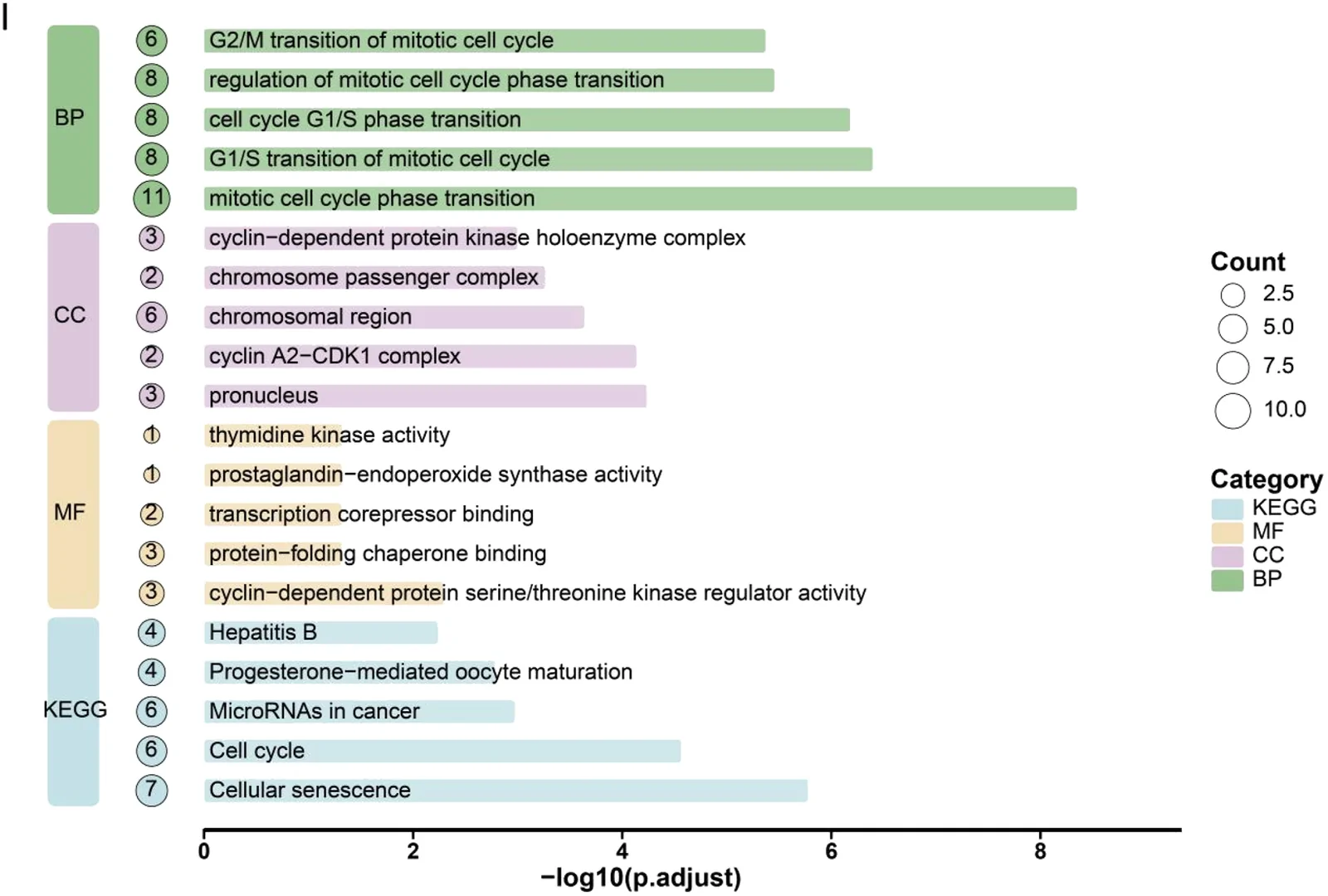
Differential transcriptomic signals and identification of key SMRGs in HCC. (A) Volcano plot summarizing DEGs between tumor and non-tumor tissues. (B) Determination of the WGCNA soft-thresholding power based on scale-free topology fit and mean connectivity. (C) Gene dendrogram with module assignment obtained by dynamic tree cutting and subsequent module merging. (D) Module eigengene–trait correlation heatmap linking co-expression modules to tumor/normal status. (E) Relationship between module membership (MM) and gene significance (GS) for genes within the turquoise module. (F) GO enrichment of turquoise-module key genes across biological process, cellular component, and molecular function categories. (G) KEGG pathway enrichment of turquoise-module key genes. (H) Overlap of DEGs, WGCNA turquoise-module key genes, and SMRGs illustrated by a venn diagram. (I) Functional annotation (GO and KEGG) of the intersected DESMRGs.

### Machine-learning–based feature selection and diagnostic model evaluation

To prioritize diagnostic biomarkers from the 24 DESMRGs, multiple machine-learning approaches were applied for feature selection. At the selected λ value, the LASSO procedure yielded a parsimonious panel of 18 genes (Figure 2A and B). In parallel, RF feature selection based on the criterion MeanDecreaseGini >1 identified 18 important genes (Figure 2C and D). Additionally, XGBoost ranked the top 5 genes as the most informative features (Figure 2E), including NTF3, LIFR, PVALB, CDKN2A, and TEK. Intersecting the candidate sets derived from LASSO, RF, and XGBoost yielded four shared feature genes – CDKN2A, LIFR, NTF3, and PVALB (Figure 2F). Expression comparisons demonstrated that CDKN2A was upregulated in tumor samples, whereas LIFR, NTF3, and PVALB were downregulated relative to normal tissues (Figure 2G). To characterize gene-specific functional programs, we performed GSEA separately for each diagnostic marker and reported the significantly enriched pathways in Supplementary Figure S1 (p<0.05). In the training cohort, ROC analysis showed strong diagnostic performance for individual genes (AUCs: CDKN2A=0.936, LIFR=0.984, NTF3=0.989, PVALB=0.977), and the combined four-gene model achieved an AUC of 0.996 (Figure 2H). To enable individualized probability estimation, we translated the four-biomarker classifier into a nomogram (Figure 2I). The DCA results supported a meaningful net benefit for the nomogram across clinically relevant threshold probabilities (Figure 2J). Calibration curves showed minimal deviation between estimated and actual risks (Figure 2K). External validation in GSE14520 confirmed robust accuracy (AUC=0.962; Figure 2L), while CDKN2A, LIFR, NTF3, and PVALB yielded AUCs of 0.827, 0.844, 0.915, and 0.702, respectively (Figure 2L).

**Figure 2:**
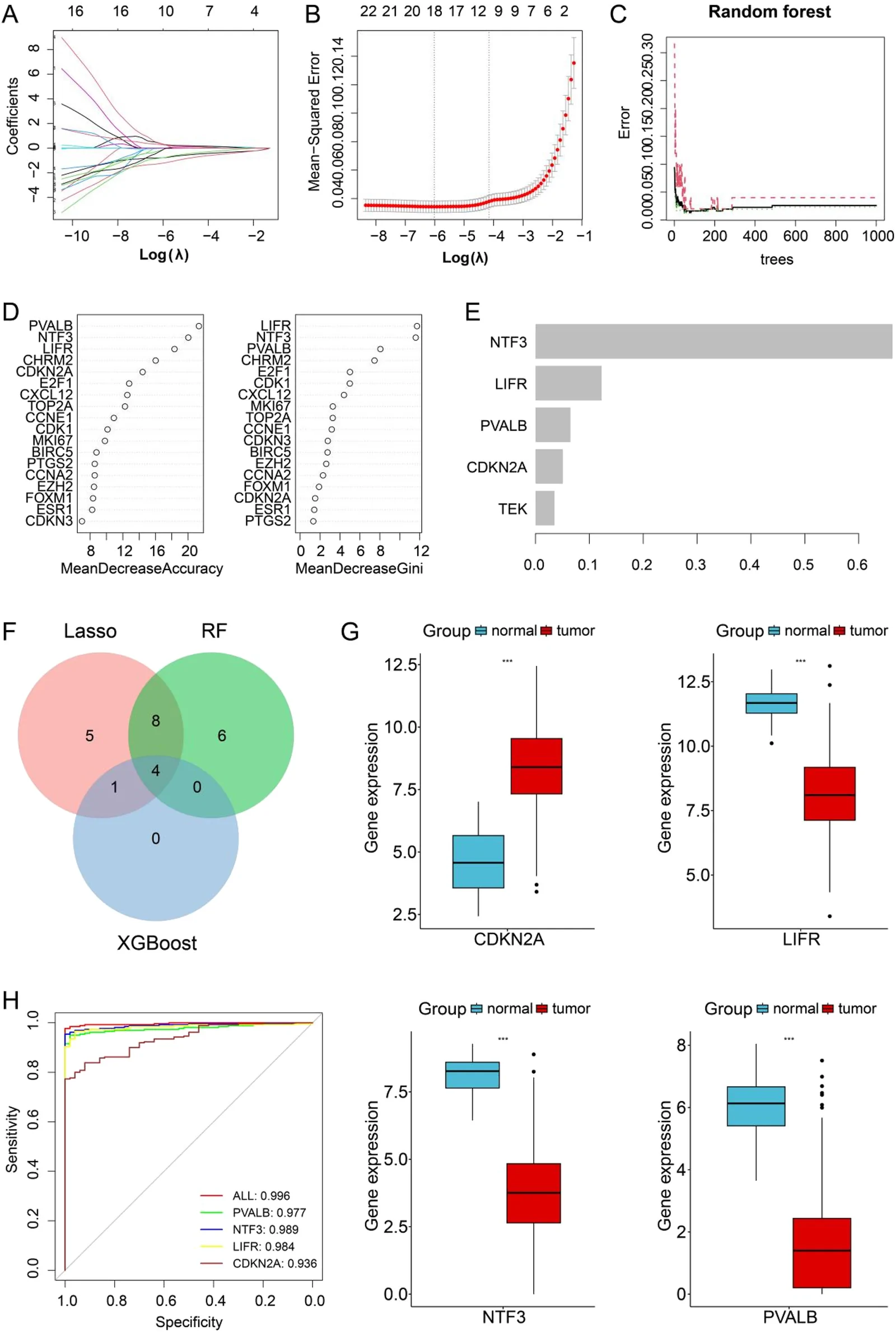

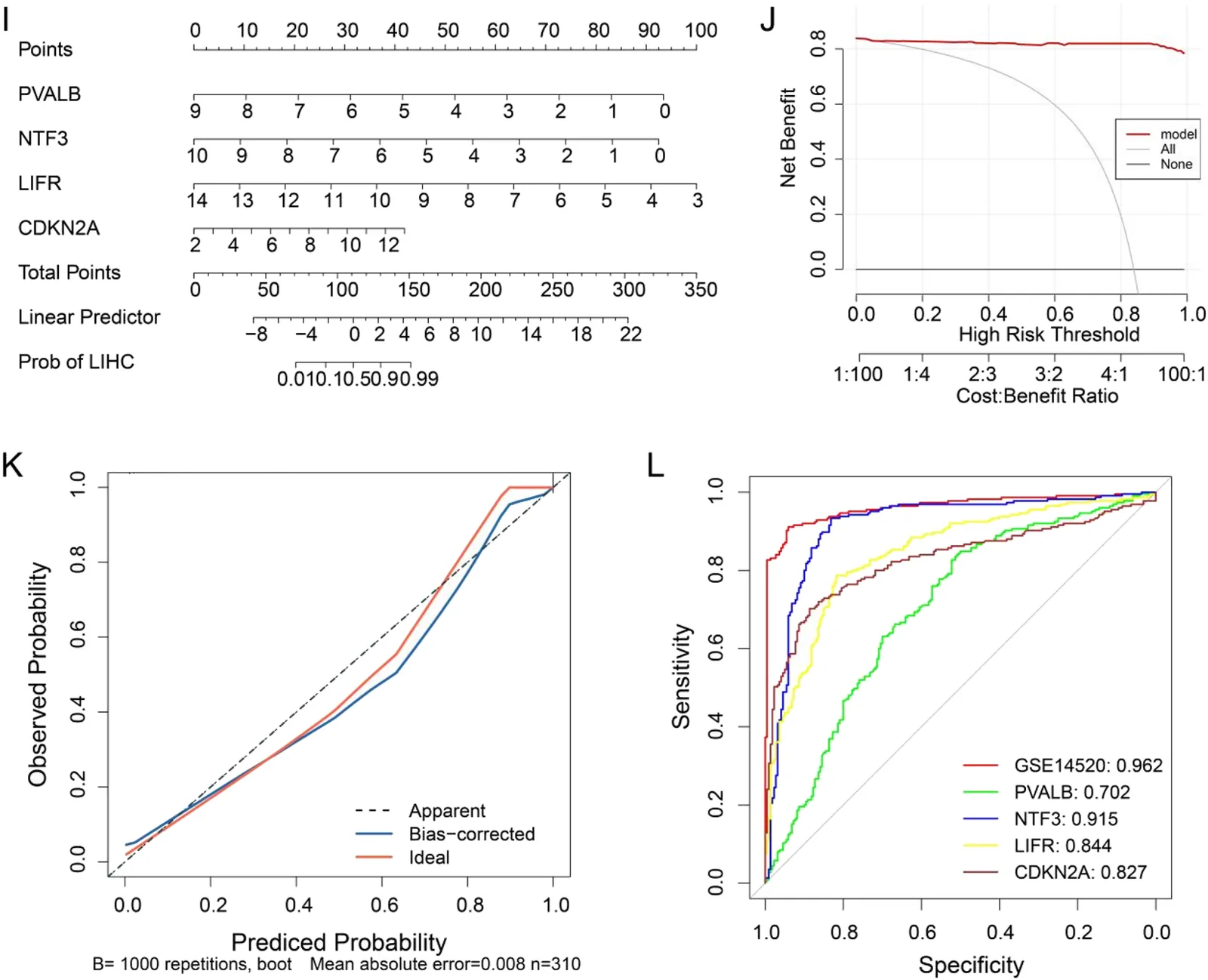
Machine-learning workflow for feature prioritization and development of a four-gene diagnostic signature for HCC. (A) LASSO coefficient trajectories for candidate genes plotted against log(λ). (B) Ten-fold cross-validation curve for LASSO with selection of the optimal penalty parameter. (C) RF out-of-bag error curve across different numbers of trees. (D) RF feature importance ranked by MeanDecreaseAccuracy and MeanDecreaseGini. (E) XGBoost importance scores highlighting the top-ranked contributors. (F) Venn diagram summarizing the intersection of features selected by LASSO, RF, and XGBoost, yielding four shared genes (CDKN2A, LIFR, NTF3, and PVALB). (G) Boxplots comparing expression levels of the four diagnostic genes between tumor and non-tumor tissues. (H) ROC curves assessing the diagnostic performance of the combined four-gene model and each single gene in the training cohort. (I) Nomogram derived from the four-gene model for individualized probability estimation of HCC. (J) DCA evaluating net clinical benefit across a range of threshold probabilities. (K) Calibration plot assessing agreement between predicted and observed probabilities for the nomogram. (L) ROC curves in the external GSE14520 cohort validating the diagnostic accuracy of the four-gene model and individual genes.

### Immune landscape analysis

We profiled immune-cell infiltration and immune functional activity using ssGSEA and observed a broad attenuation of immune signatures in tumors relative to non-tumor tissues (Figure 3A). Normal tissues exhibited relatively higher inferred immune-related signatures compared with tumors, with increases were most evident for B cells, CD8+ T cells, NK cells, plasmacytoid dendritic cells (pDCs), and T follicular helper (Tfh) cells. In line with these cellular differences, several immune-related programs were also more active in normal tissues, such as inflammation-promoting signaling, MHC class I–associated pathways, and type I/II interferon response signatures (Figure 3A). To obtain cell-fraction estimates, we further performed CIBERSORT deconvolution (Figure 3B). CIBERSORT suggested differences in inferred cell-type distributions between tumors and normal tissues. T cells (Tregs), Th cells, and resting dendritic cells were increased in tumors, whereas plasma cells, resting NK cells, and M2 macrophages were estimated to be more abundant in normal samples, pointing to immune-cell redistribution within the TME. MCP-counter profiling captured broad alterations across both immune and stromal components between tumor and non-tumor tissues. Notably, tumors displayed lower scores for cytotoxic lymphocytes, B-lineage cells, NK cells, and the monocytic lineage (Figure 3C), further supporting differences in inferred immune composition between groups. To explore the immunological relevance of the diagnostic signature, Pearson correlation analyses were performed between immune infiltration/immune function signatures and the four model genes (CDKN2A, LIFR, NTF3, and PVALB), and p values were adjusted for multiple testing using the Benjamini–Hochberg method. Significant gene–immune associations were observed across a broad panel of immune-related features, including antigen presentation– and T-cell–related functions (e.g., MHC class I, T-cell co-stimulation/co-inhibition, and APC co-stimulation/co-inhibition) as well as interferon-response signatures (Figure 3D–G). Overall, these findings are based on enrichment scores derived from bulk transcriptomic data and should be interpreted as inferred immune activity rather than direct measurements.

**Figure 3:**
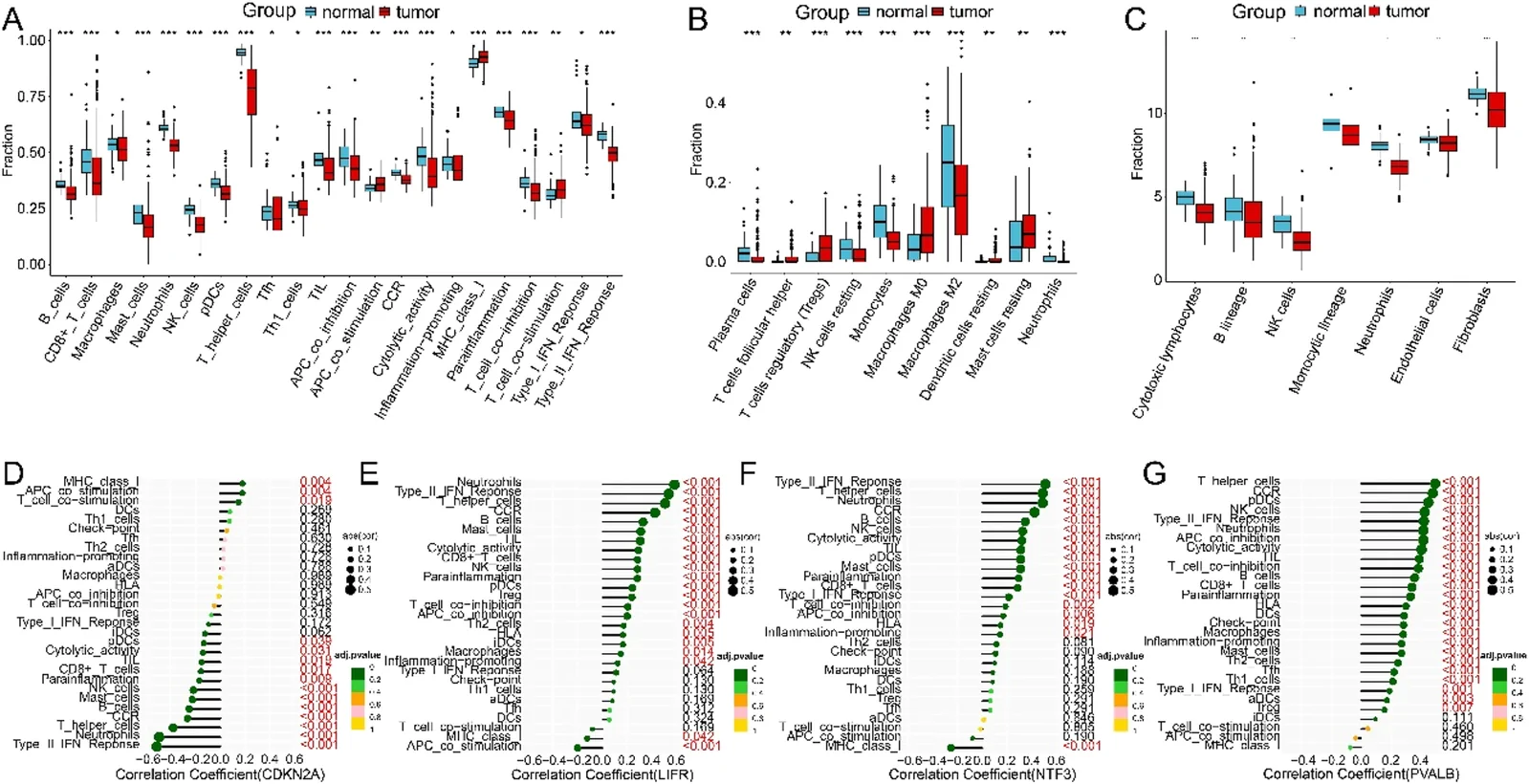
Immune landscape differences between tumor and normal tissues and their associations with the diagnostic genes. (A) ssGSEA profiling of immune-cell signatures and immune functional activities in tumor vs. non-tumor samples. (B) CIBERSORT-estimated immune cell fractions in normal vs. tumor tissues. (C) MCP-counter–based estimation of immune and stromal population scores in normal and tumor samples. (D–G) Pearson correlation analyses between immune signatures and the four diagnostic genes: (D) CDKN2A, (E) LIFR, (F) NTF3, and (G) PVALB.

### Prognostic DESMRGs and succinate metabolism–related molecular subtypes

Univariate survival regression analysis of the DESMRGs identified 12 genes significantly associated with prognosis (Figure 4A), including CDK1, BIRC5, TOP2A, E2F1, EZH2, MKI67, CCNA2, RECQL4, CDKN2A, ESR1, CDC25A, and TEK. Most genes were associated with increased risk (HR>1), while ESR1 and TEK were linked to reduced risk (HR<1) (Figure 4A). Consistent with these patterns, expression profiling demonstrated that CDK1, BIRC5, TOP2A, E2F1, EZH2, MKI67, CCNA2, RECQL4, CDKN2A, and CDC25A were markedly upregulated in tumor tissues compared with normal controls, while ESR1 and TEK were downregulated in tumors (Figure 4B). Correlation analysis further revealed strong positive co-expression among the proliferation- and cell cycle–related genes, whereas TEK and ESR1 exhibited broadly negative correlations with this proliferative gene cluster (Figure 4C). The genomic positions of the 12 prognosis-related genes were mapped in a circos diagram (Figure 4D). Using the expression patterns of these genes, ConsensusClusterPlus partitioned HCC cases into two molecular subtypes (Figure 4E–G). Model selection favored k=2, supported by the consensus matrix together with the Cumulative distribution function (CDF) curves and the delta area plot (Figure 4E–G). PCA provided an orthogonal validation of this classification, showing clear separation of the two subtypes (PC1=65.3 %, PC2=9.3 %; Figure 4H). Kaplan–Meier analysis further revealed significantly worse overall survival in subtype 1 than in subtype 2 (log-rank p<0.0041; Figure 4I). To interpret the biological basis of these subtypes, we identified subtype-associated DEGs and performed functional enrichment analysis. The enriched signals were dominated by cell-division programs (e.g., chromosome segregation and nuclear division), while KEGG pathways additionally highlighted Cell cycle and metabolism-related processes, including drug/xenobiotic metabolism by cytochrome P450, retinol metabolism, and bile secretion (Figure 4J).

**Figure 4:**
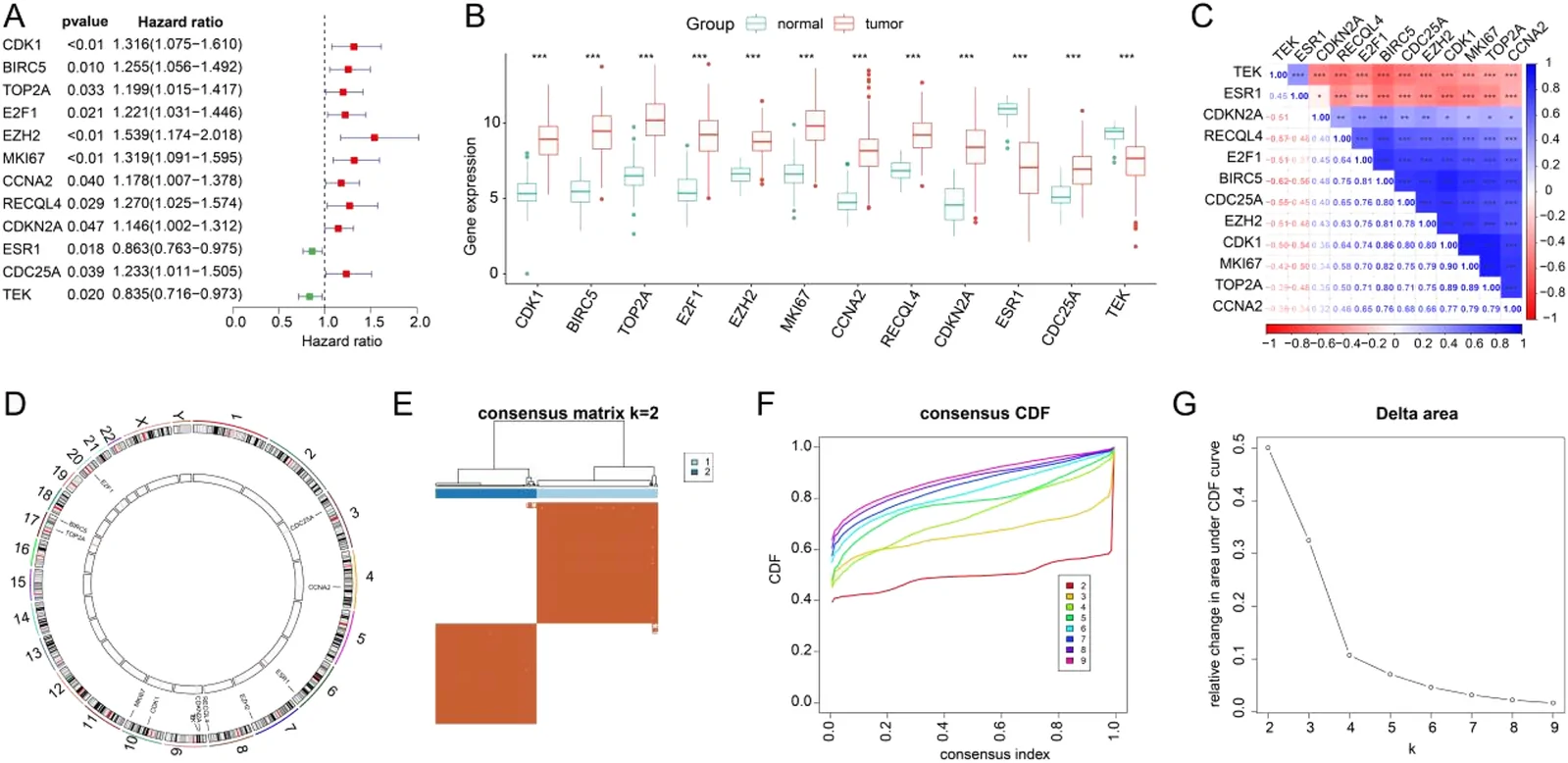

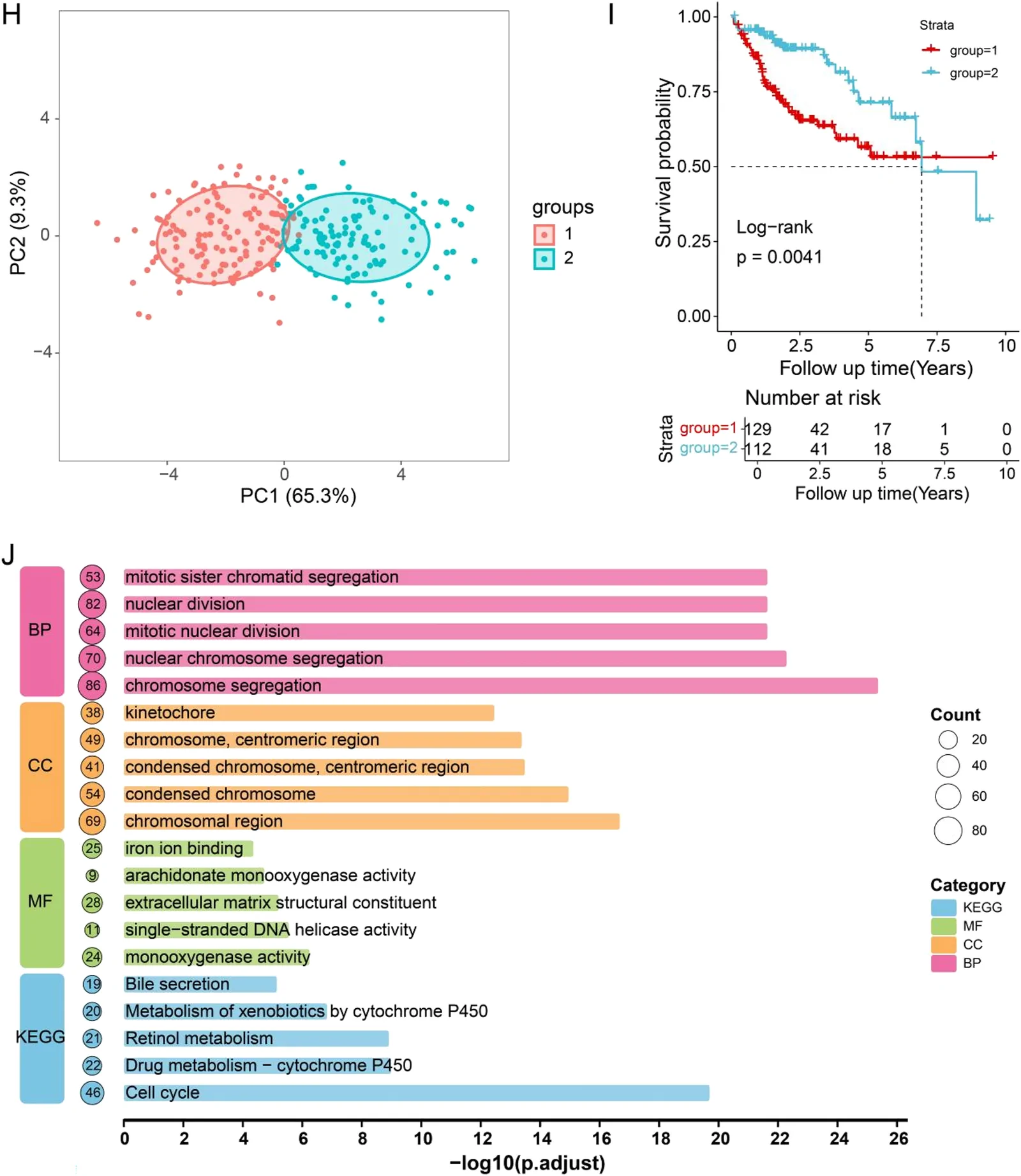
Prognosis-associated DESMRGs and consensus clustering–defined molecular subtypes in HCC. (A) Forest plot summarizing univariate Cox regression results for DESMRGs. (B) Expression distribution of prognosis-associated genes in tumor vs. non-tumor tissues (boxplots). (C) Pairwise expression correlation matrix among the prognosis-associated genes (heatmap). (D) Chromosomal mapping of prognosis-associated genes displayed by a circos plot. (E) Consensus matrix generated by ConsensusClusterPlus for k=2. (F) CDF curves of the consensus index across candidate cluster numbers. (G) Delta area plot quantifying changes in the CDF area to support the optimal k. (H) PCA projection illustrating separation of the two molecular subtypes. (I) Kaplan–Meier overall survival comparison between the two subtypes with the log-rank p value. (J) Functional enrichment of subtype-associated DEGs by GO (BP/CC/MF) and KEGG analyses.

### Subtyping-based prognostic model construction and validation

Univariate Cox screening of subtype-associated genes prioritized 26 candidates with significant survival associations (Figure 5A; Supplementary Table S1). To derive a parsimonious signature, we applied LASSO penalization and selected the optimal λ, which retained 16 genes for downstream modeling (Figure 5B and C; Supplementary Table S2). These candidates were then entered into multivariate Cox regression, and a six-gene prognostic signature was established, comprising LCAT, EPO, GLDN, MAGEA6, SGPP2, and FAM171A1 (Figure 5D; Supplementary Table S3). Using this signature, each patient in the training cohort (n=241) was assigned a risk score and stratified into high- and low-risk groups by the median score. Time-dependent ROC analysis demonstrated AUCs of 0.803, 0.720, and 0.803 at 1, 3, and 5 years, respectively (Figure 5E). Kaplan–Meier analysis showed significantly worse overall survival in the high-risk group (p<0.0001; Figure 5F), and the distribution of risk scores together with survival status is presented in Figure 5G. External validation in the GSE76427 cohort confirmed the predictive performance of the signature, with AUCs of 0.729, 0.714, and 0.685 at 1, 3, and 5 years, respectively (Figure 5H). Consistently, the high-risk group exhibited poorer survival than the low-risk group (p=0.018; Figure 5I), and risk score and survival status distributions are shown in Figure 5J. Expression profiling indicated that LCAT, EPO, and SGPP2 were significantly lower in tumor tissues than in normal controls, whereas GLDN and MAGEA6 were higher in tumors; FAM171A1 showed no significant difference (Figure 5K). In risk-stratified comparisons, EPO, GLDN, MAGEA6, and SGPP2 were upregulated in the high-risk group, while LCAT and FAM171A1 were downregulated (Figure 5L). Kaplan–Meier analyses further supported the prognostic relevance of each signature gene: higher expression of EPO, GLDN, MAGEA6, and SGPP2 was associated with poorer survival, whereas higher expression of LCAT and FAM171A1 predicted more favorable outcomes (Figure 5M).

**Figure 5:**
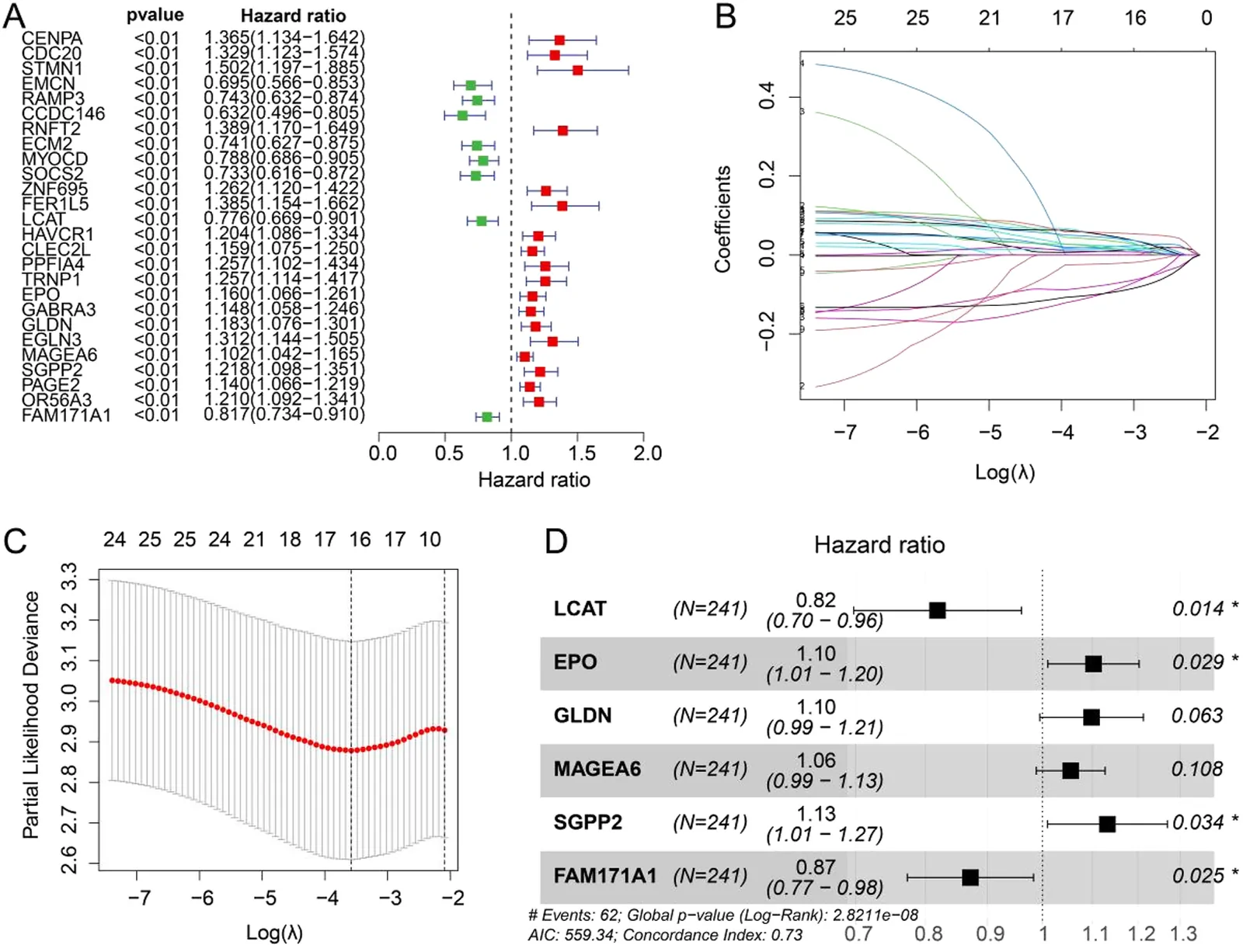

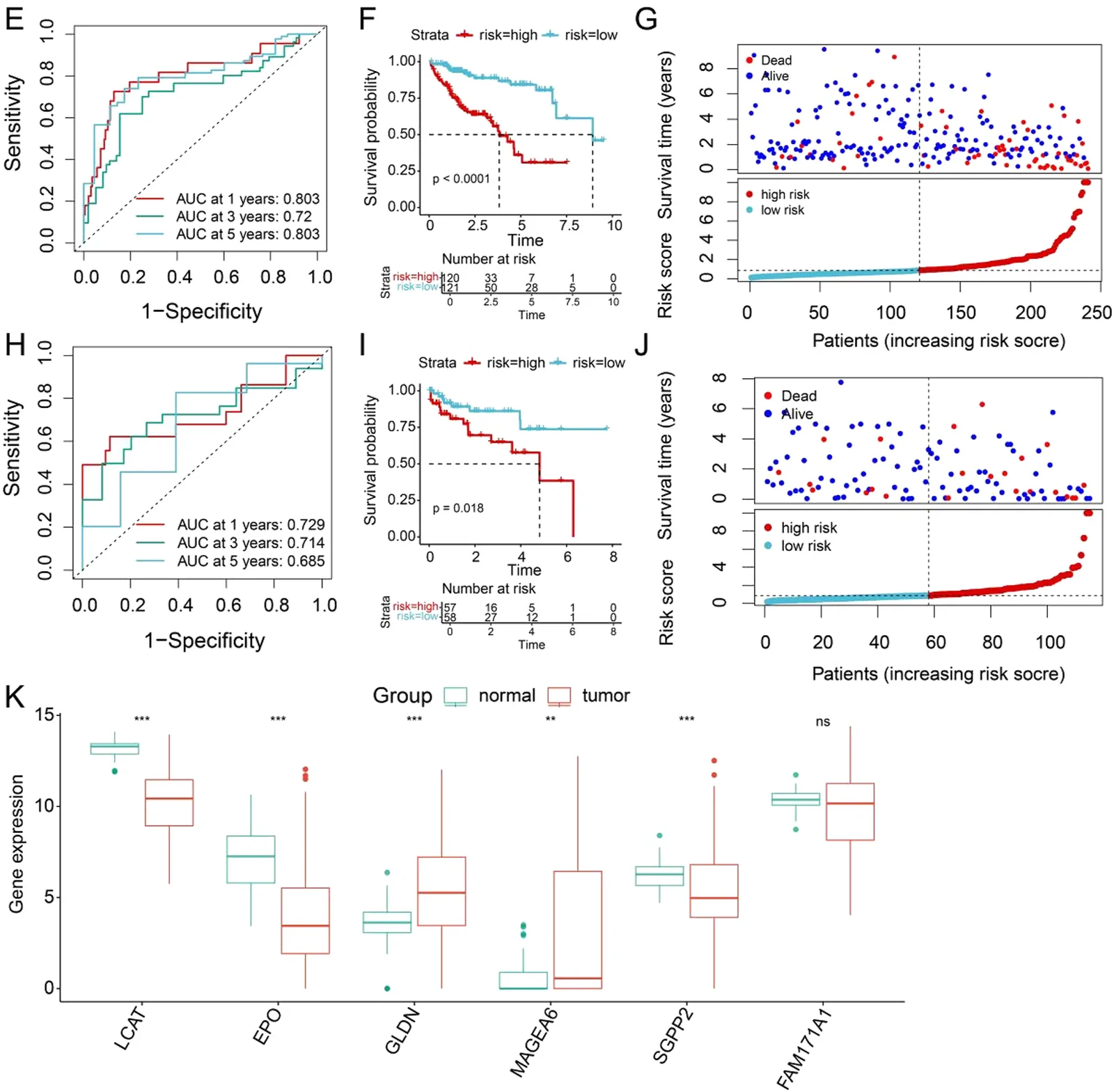

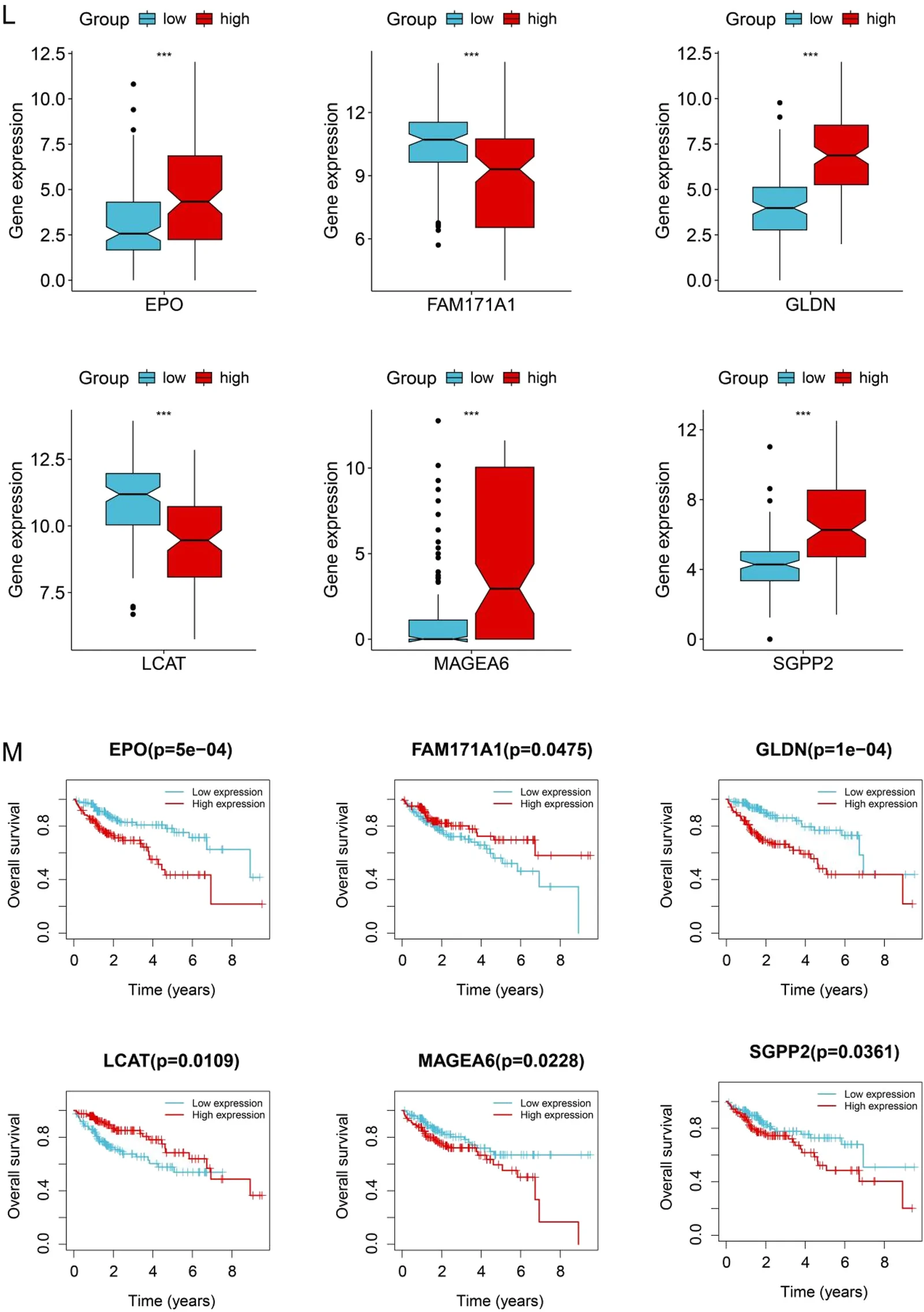
Development and validation of a subtype-derived six-gene prognostic signature in HCC. (A) Univariate Cox screening of subtype-associated DEGs presented as a forest plot (HRs with 95 % CIs and p values). (B) LASSO coefficient paths for candidate prognostic genes across log(λ). (C) LASSO cross-validation curve (partial likelihood deviance) with the optimal penalty parameter highlighted. (D) Multivariable Cox regression forest plot defining the final six-gene signature in the training cohort (n=241). (E) Time-dependent ROC curves at 1, 3, and 5 years in the training cohort. (F) Kaplan–Meier overall survival stratified by the risk score (high vs. low) in the training cohort. (G) Risk score distribution with corresponding survival status in the training cohort. (H) Time-dependent ROC curves at 1, 3, and 5 years in the external validation cohort (GSE76427). (I) Kaplan–Meier survival comparison between high- and low-risk groups in the validation cohort. (J) Risk score distribution and survival status in the validation cohort. (K) Tumor vs. non-tumor expression of the six signature genes (boxplots). (L) Expression of the six signature genes across risk groups (high vs. low; boxplots). (M) Gene-level Kaplan–Meier analyses for each signature gene (high vs. low expression).

### Risk-group enrichment analysis

GSEA revealed distinct pathway patterns between the two groups (Figure 6A). Specifically, the high-risk group showed enrichment of cell-cycle–related programs, including mitotic cell cycle phase transition and regulation of mitotic cell cycle phase transition, whereas the low-risk group was preferentially enriched for metabolic processes such as fatty acid beta-oxidation and fatty acid catabolic process. Consistently, functional annotation of the DEGs between the two risk groups further highlighted cell-division–associated biological processes. GO enrichment was dominated by terms related to chromosome segregation and mitosis, including mitotic sister chromatid segregation, mitotic nuclear division, nuclear division, and nuclear chromosome segregation (Figure 6B). KEGG pathway analysis also indicated significant enrichment in Cell cycle and multiple metabolism-related pathways, including retinol metabolism, bile secretion, and drug/xenobiotic metabolism by cytochrome P450, among others (Figure 6C).

**Figure 6:**
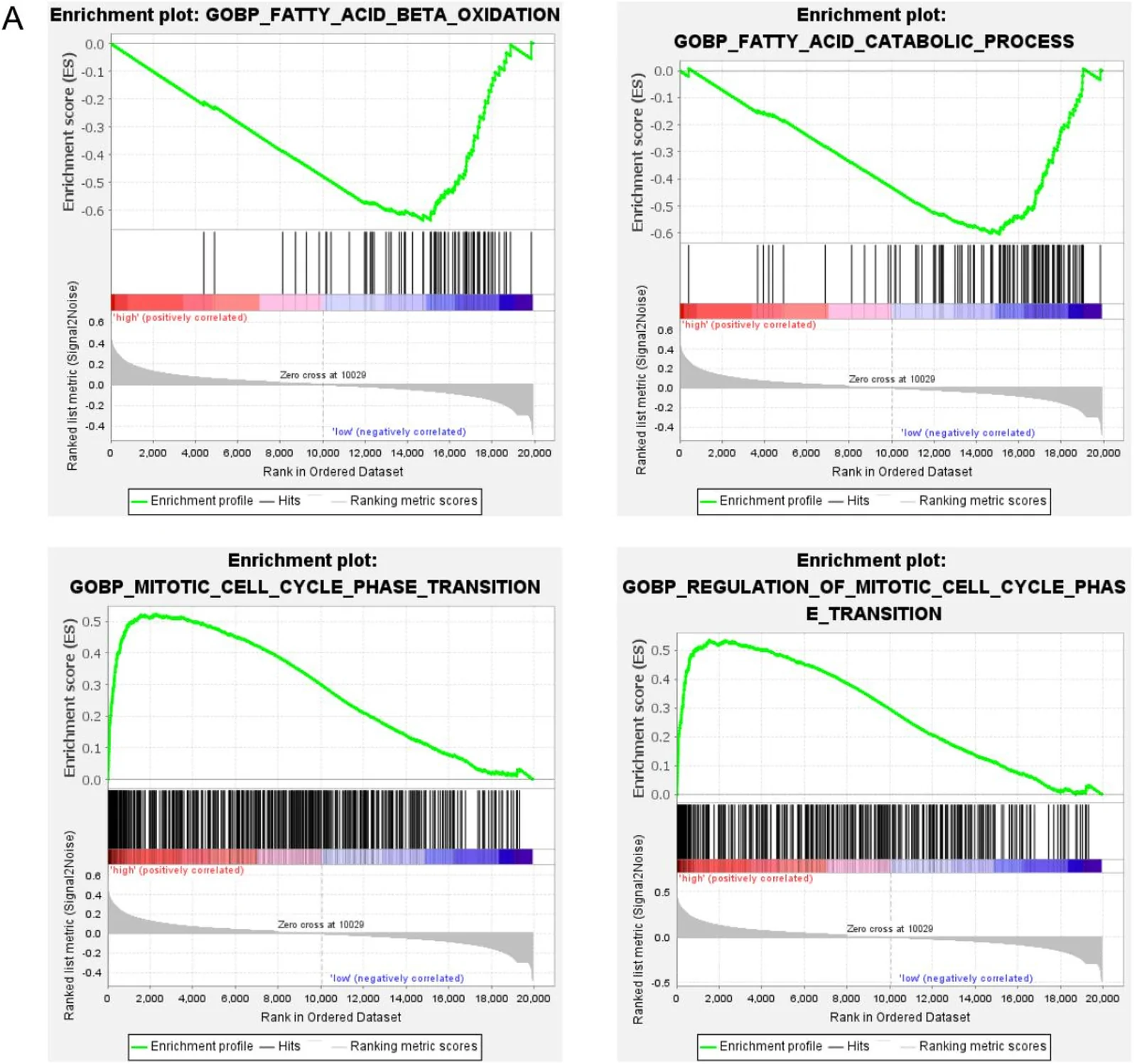

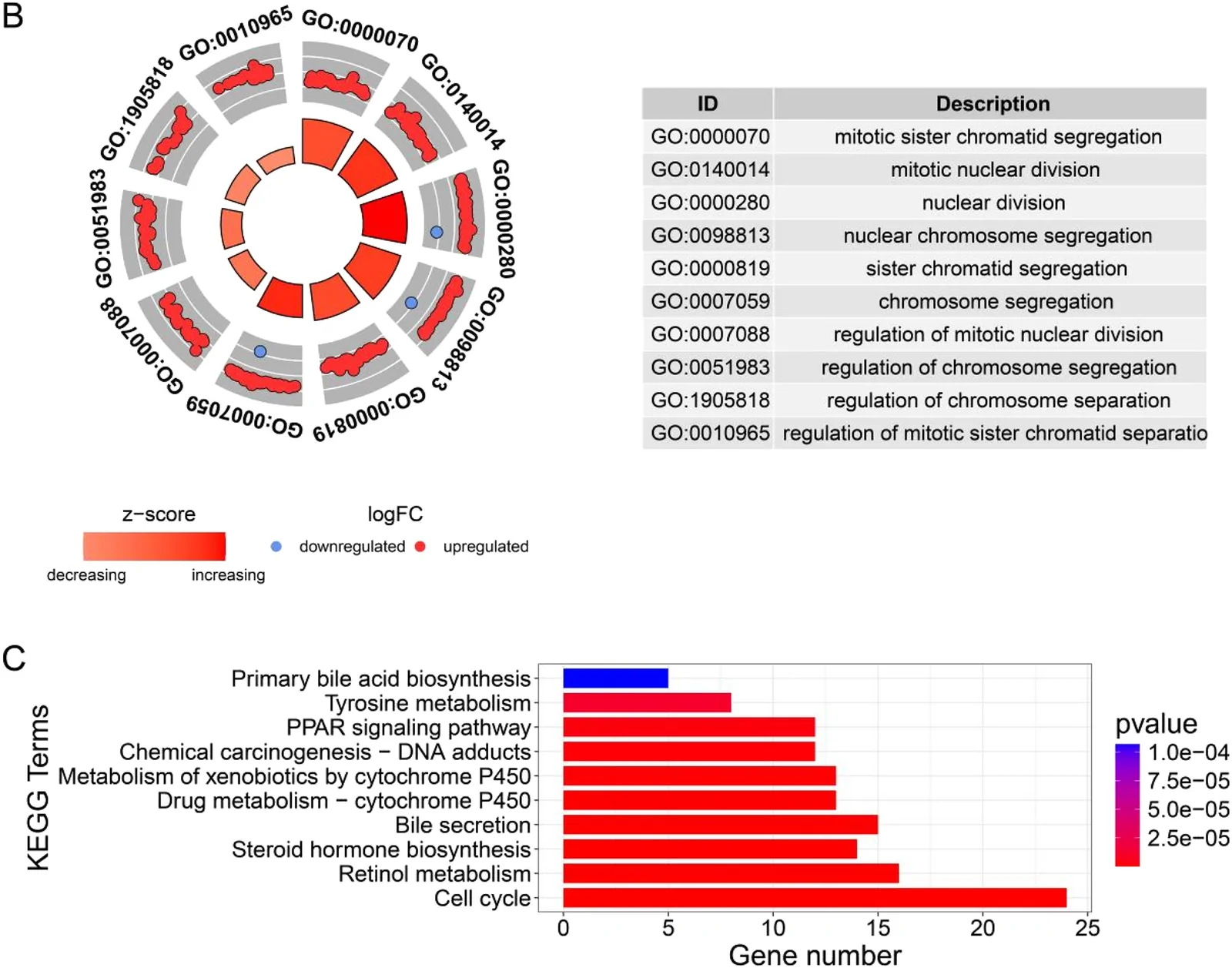
Functional pathway differences associated with the risk stratification. (A) GSEA results highlighting pathways differentially enriched between the high- and low-risk groups. (B) GO functional enrichment of DEGs identified from the high-vs. low-risk comparison. (C) KEGG pathway enrichment of DEGs derived from the high-vs. low-risk comparison.

### Prognostic value of the risk score after adjustment for clinical variables and subgroup analyses

Univariate Cox regression incorporating the risk score and clinicopathological variables showed that the riskScore was significantly associated with overall survival, whereas age, gender, stage, and T stage were not statistically significant in this dataset (Figure 7A). In multivariate Cox analysis, the riskScore remained significantly associated with overall survival after adjustment for age, gender, and stage (Figure 7B). A nomogram integrating riskScore and clinical variables retained in the multivariate model (gender and stage) was constructed to estimate 1-, 3-, and 5-year survival probabilities (Figure 7C). DCA demonstrated a favorable net benefit of the nomogram across a range of threshold probabilities for predicting 1-, 3-, and 5-year survival, supporting its potential clinical utility (Figure 7D). Calibration plots further indicated good agreement between predicted and observed survival at 1, 3, and 5 years (Figure 7E). Collectively, these results suggest that the six-gene–derived risk score retains prognostic value for overall survival after adjustment for available clinical variables. In addition, riskScore distributions were compared across clinical subgroups (age, gender, stage, and T stage) using violin plots, showing significant differences for gender, stage, and T stage but not for age (Supplementary Figure S2). Stratified Kaplan–Meier analyses consistently indicated poorer overall survival in the high-risk subgroup across age (≤65 vs. >65), gender (female vs. male), stage (I vs. II), and T stage (T1 vs. T2) strata (Supplementary Figure S3). Clinical characteristics between the high- and low-risk groups are summarized in Table 1.

**Figure 7:**
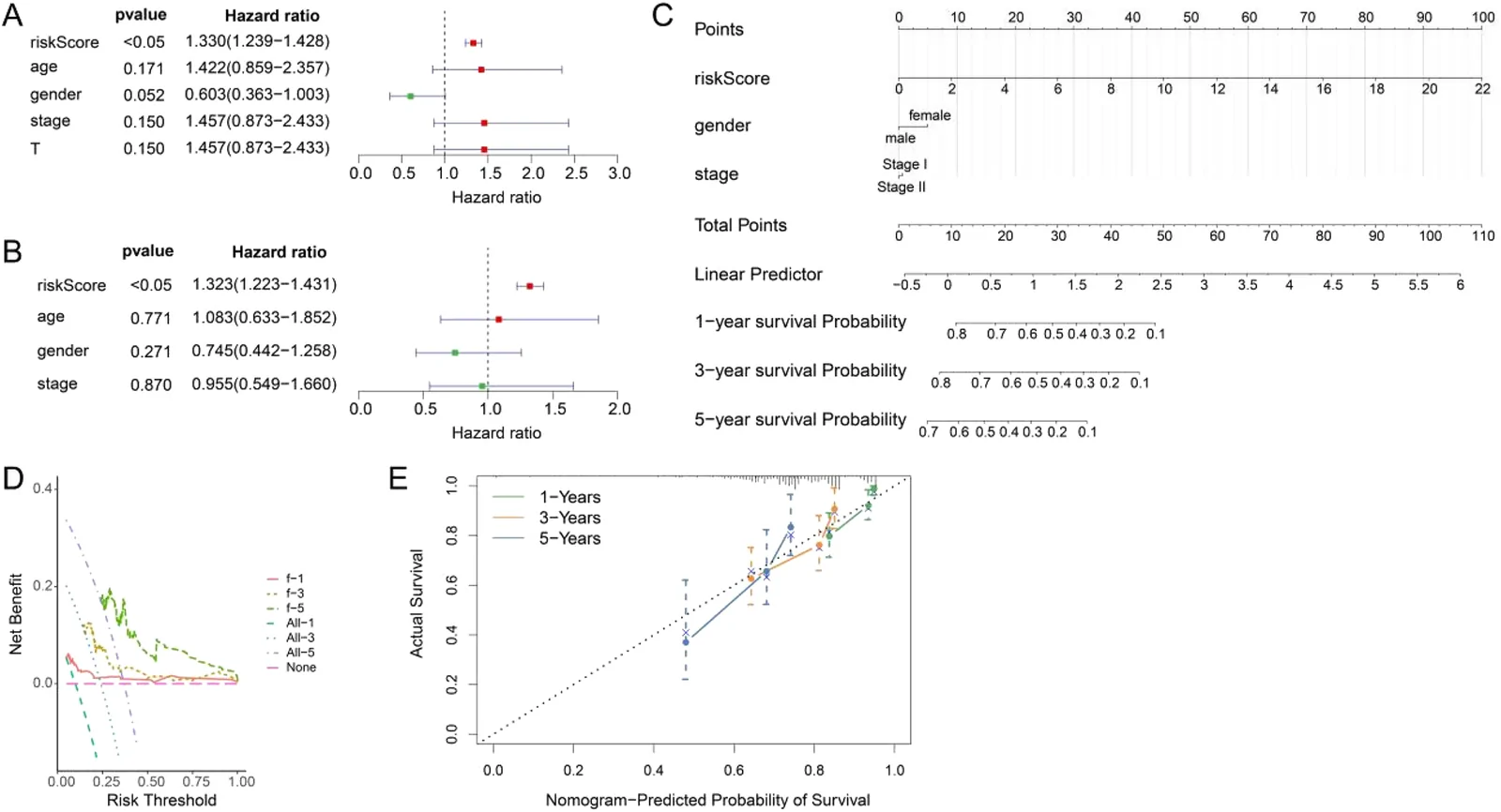
Independence assessment and clinical utility of the risk score–based prognostic model. (A) Univariate Cox analysis of overall survival for the risk score and clinicopathological variables (age, sex, stage, and T category). (B) Multivariable Cox model including the risk score and clinical covariates, showing its association with overall survival after adjustment. (C) Nomogram combining the risk score with Cox-retained clinical factors to estimate individualized survival. (D) DCA quantifying the net benefit of the nomogram across clinically relevant thresholds. (E) Calibration curves assessing agreement between predicted and observed survival at 1, 3, and 5 years.

**Table 1:** Summary of clinicopathological characteristics between the high- and low-risk groups.

Name	Levels	High (n=120)	Low (n=121)	p-Value
Fustat	0	74 (61.7 %)	105 (86.8 %)	<0.001
	1	46 (38.3 %)	16 (13.2 %)	
Age	Mean ± SD	60.6 ± 13.1	58.9 ± 11.5	0.266
Gender	Female	38 (31.7 %)	33 (27.3 %)	0.544
	Male	82 (68.3 %)	88 (72.7 %)	
Stage	Stage I	72 (60 %)	91 (75.2 %)	0.017
	Stage II	48 (40 %)	30 (24.8 %)	
T	T1	72 (60 %)	90 (74.4 %)	0.028
	T2	48 (40 %)	30 (24.8 %)	
	Unknown	0 (0 %)	1 (0.8 %)	
n	n0	85 (70.8 %)	87 (71.9 %)	0.967
	Unknown	35 (29.2 %)	34 (28.1 %)	
M	M0	87 (72.5 %)	90 (74.4 %)	0.854
	Unknown	33 (27.5 %)	31 (25.6 %)	

### Risk-stratified immune landscape and predicted immunotherapy responsiveness

To characterize immune differences associated with the prognostic signature, ssGSEA revealed significant risk-dependent variations in multiple immune cell and immune function scores (Figure 8A). Overall, compared with the low-risk group, the high-risk group exhibited a more immunosuppressive-like immune profile, with higher scores for activated dendritic cells (aDCs) and macrophages, as well as increased Tfh cells and Tregs. In terms of immune functions, the high-risk group also displayed higher scores for APC co-inhibition and MHC class I–related signatures. In contrast, the low-risk group showed higher scores for mast cells and the Type II IFN response. Consistent with ssGSEA, CIBERSORT further indicated differential immune-cell composition between risk groups (Figure 8B). Notably, Tfh cells and Tregs displayed higher inferred fractions in the high-risk group, whereas monocytes and mast cells resting showed comparatively higher infiltration in the low-risk group, suggesting a more immune-permissive microenvironment in low-risk tumors. Given the observed immune heterogeneity, we next examined immune checkpoint profiles. Most immune checkpoint–related genes were expressed at higher levels in the high-risk group than in the low-risk group (Figure 8C), indicating that the six-gene risk score was associated with a generally upregulated checkpoint landscape. We then evaluated whether the risk score could reflect immunotherapy responsiveness using the IMvigor210 cohort treated with anti–PD-L1 therapy. Patients were categorized as non-responders (NR; SD/PD) or responders (R; CR/PR). The low-risk group exhibited a higher objective response rate than the high-risk group (26.8 vs. 18.8 %, p<0.001; Figure 8D), suggesting a potential association between the risk score and response patterns. In parallel, TIDE analysis showed significantly higher TIDE scores in the high-risk group than in the low-risk group (Wilcoxon test, p=2.1 × 10^−6^; Figure 8E), indicating a potential difference in immune evasion–related features between risk groups. Finally, correlation analysis integrating CIBERSORT-inferred immune infiltration with model genes demonstrated broad associations between immune cell fractions and the expression of the six signature genes (LCAT, EPO, GLDN, MAGEA6, SGPP2, and FAM171A1) (Figure 8F).

**Figure 8:**
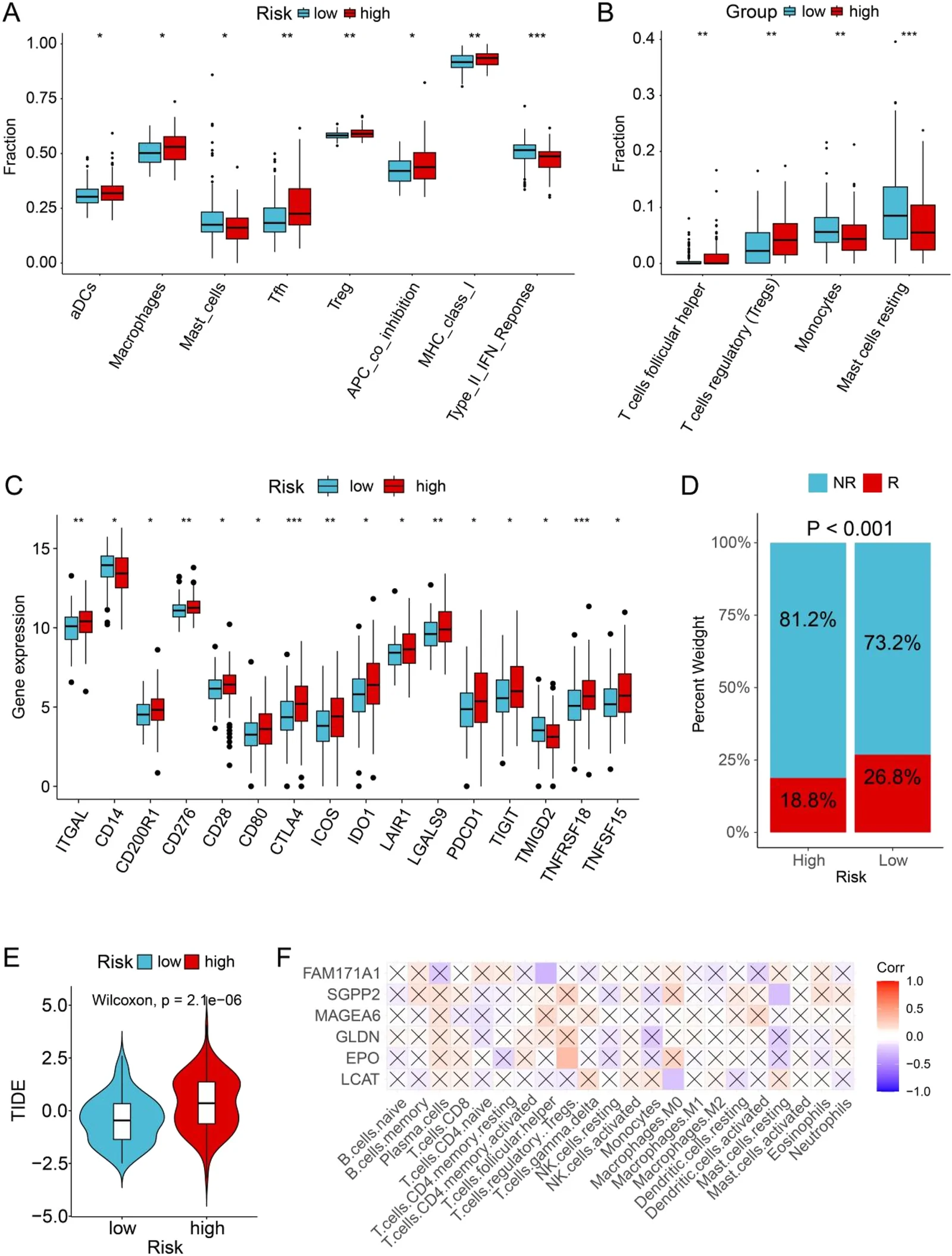
Risk-stratified immune features and immunotherapy-response prediction associated with the six-gene prognostic signature. (A) ssGSEA-derived immune-cell infiltration signatures and immune functional scores across low- and high-risk groups. (B) CIBERSORT deconvolution of immune-cell fractions stratified by risk group. (C) Immune checkpoint–related gene expression patterns in low-vs. high-risk patients. (D) IMvigor210 cohort analysis (anti–PD-L1 treated) for risk score–associated immunotherapy response. (E) TIDE score distribution in low- and high-risk groups (violin plot; Wilcoxon test). (F) Pearson correlation heatmap linking six signature genes (LCAT, EPO, GLDN, MAGEA6, SGPP2, and FAM171A1) with CIBERSORT-inferred immune-cell fractions.

### Somatic mutation landscapes differed between the two risk strata

In the high-risk group, the oncoplot showed that 106 of 116 samples (91.38 %) harbored alterations among the top 20 mutated genes, with TP53 (43 %), TTN (24 %), MUC16 (18 %), and CTNNB1 (16 %) being the most frequently mutated genes (Supplementary Figure S4A). In the low-risk group, 104 of 118 samples (88.14 %) exhibited alterations, and CTNNB1 (32 %), TTN (24 %), ALB (19 %), and TP53 (16 %) were the leading mutated genes (Supplementary Figure S4B). Consistently, TMB was significantly higher in the high-risk group than in the low-risk group (Wilcoxon test, p=0.024; Supplementary Figure S4C). Drug response analyses based on transcriptomic inference further suggested risk-associated differences in predicted therapeutic response patterns. Compared with the low-risk group, the high-risk group exhibited significantly lower predicted IC50 values for 5-fluorouracil (p=6.8 × 10^−4^), cisplatin (p=9.9 × 10^−6^), and XL-184 (p=2.6 × 10^−3^), whereas sorafenib showed a modest but significant increase in predicted IC50 in the high-risk group (p=0.046) (Supplementary Figure S4D–G). In addition, correlation analyses indicated associations between gene expression and inferred drug response: SGPP2 expression was negatively associated with cisplatin IC50 (r=−0.285, p=0.027) (Supplementary Figure S4H); LCAT expression associated negatively with sunitinib IC50 (r=−0.258, p=0.046) but positively with gemcitabine IC50 (r=0.371, p=0.004) (Supplementary Figure S4I–J); and FAM171A1 expression was inversely associated with fluorouracil IC50 (r=−0.297, p=0.021) (Supplementary Figure S4K; Supplementary Table S4).

## Discussion

In this study, we demonstrated that gene expression signatures related to succinate metabolism may serve as effective biomarkers for early-stage HCC diagnosis and prognosis. Using a machine-learning approach on transcriptomic datasets, we identified a diagnostic gene panel encompassing several succinate metabolism–associated genes, and we developed a SMRG-based prognostic risk score that may stratify early-stage HCC patients into distinct risk groups. Overall, our findings highlight that dysregulation of succinate metabolic pathways may contribute to hepatocarcinogenesis, while also representing a measurable molecular signature with potential clinical utility in HCC management.

The SMRG-derived gene panel was able to distinguish early-stage HCC from non-tumor liver tissue with robust performance. Notably, some genes in the panel have known links to cancer metabolism and might explain the panel’s diagnostic power. For example, one of the top contributors was SGPP2 (sphingosine-1-phosphate phosphatase 2), an enzyme that degrades sphingosine-1-phosphate (S1P) [30]. S1P is a bioactive lipid that promotes cell proliferation and angiogenesis; altered sphingolipid metabolism has been observed in HCC and can modulate immune cell trafficking [31]. Interestingly, SGPP2 was also identified as a key prognostic gene in an independent antigen-presentation gene signature for HCC, suggesting it may be a nexus between metabolism and tumor immunity, potentially reflecting broader metabolic reprogramming networks that may intersect with succinate-associated pathways [32]. Another diagnostic gene, LCAT (lecithin–cholesterol acyltransferase), is involved in cholesterol metabolism [33]. Decreased LCAT expression in our HCC samples aligns with known metabolic reprogramming in HCC toward cholesterol synthesis and uptake. Indeed, a recent study by Chang et al. found that exogenous succinate could inhibit HCC growth by down-regulating cholesterol biosynthesis via fibronectin-1 (FN1) and squalene epoxidase (SQLE) [34]. In this context, the inclusion of LCAT in our signature may reflect a compensatory disruption of cholesterol homeostasis in HCC, potentially representing a downstream manifestation of succinate-associated metabolic reprogramming that may be captured by the SMRG axis and could be exploited diagnostically. We also identified less-characterized genes like FAM171A1 (also known as Astroprincin) as discriminators of HCC. FAM171A1 has no direct metabolic function but has been linked to cell adhesion and cytoskeletal dynamics; its overexpression was recently reported as a marker of poor prognosis in colorectal cancer [35]. In our study, its upregulation in HCC tumor tissue (vs. normal liver) may indicate enhanced cell motility and a shift toward an invasive phenotype even at early stages, which could represent downstream cellular consequences of broader metabolic and tumor microenvironment reprogramming potentially associated with SMRG-related metabolic alterations [36]. Altogether, these findings underscore that early-stage HCC, while often clinically occult, is molecularly distinct from benign liver – particularly in genes governing metabolic and cell–microenvironment interactions – which, although not all directly involved in canonical succinate metabolism, may converge on broader metabolic reprogramming and tumor–microenvironment networks potentially linked to SMRG-associated processes. A practical implication is that a blood-based or tissue-based assay for an SMRG signature could aid early HCC detection. For instance, it may be feasible to design a qPCR panel or targeted RNA sequencing panel for a handful of succinate-related genes in liver biopsy specimens or circulating tumor RNA. Such a test, in combination with imaging, may have the potential to improve sensitivity for early HCC, pending further validation. This is especially pertinent as emerging multi-analyte biomarker panels (e.g. GALAD, which integrates AFP variants and other markers) are beginning to show promise [7]. Our results encourage further evaluation of metabolic gene panels as part of this evolving early detection toolkit.

Beyond diagnosis, we showed that SMRG signatures carry prognostic information for early-stage HCC. We constructed a risk score based on six prognostic SMRGs and found that it stratified patients into high-risk vs. low-risk groups with significantly different outcomes. The high-risk group (with an unfavorable SMRG expression pattern) had markedly shorter overall survival, consistent with more aggressive tumor biology, whereas the low-risk group had prolonged survival. This risk score remained prognostic value even after adjusting for clinicopathological factors, indicating that it captures facets of tumor behavior not apparent from standard staging (all our patients were early-stage) or histologic grading. It is noteworthy that our SMRG-based model compares favorably with previously published prognostic biomarkers for HCC. Traditional prognostic indicators for early HCC include tumor size, multifocality, microvascular invasion, and liver function parameters, but these are imperfect and often qualitative [37], 38]. In recent years, several gene expression–based prognostic signatures have been proposed for HCC [39], 40]. For example, Zhuo et al. (2024) reported an immune-metabolic 4-gene signature that predicted HCC survival with higher accuracy than immunogenetic or metabolic genes alone [41]. Another study identified a panel of antigen presentation machinery genes (including SGPP2) that correlated with prognosis and response to immunotherapy [32]. Compared with these existing models, our SMRG-based signature provides a complementary perspective by specifically focusing on succinate metabolism–associated genes, thereby capturing a distinct dimension of tumor metabolic reprogramming. While many previously reported signatures emphasize immune infiltration or general metabolic alterations, our model highlights the potential contribution of succinate-associated metabolic processes to tumor progression and patient prognosis. Notably, succinate has been increasingly recognized as an oncometabolite that may influence angiogenesis, epigenetic regulation, and metabolic rewiring in cancer [42]. These processes may collectively contribute to tumor aggressiveness and poorer clinical outcomes, providing a biological rationale for the prognostic relevance of SMRG-based signatures.

Furthermore, pathway analysis in our study revealed that high-risk tumors (with high SMRGs risk scores) were enriched for gene programs involved in cell cycle progression and glycolysis, whereas low-risk tumors showed upregulation of fatty acid β-oxidation and bile acid metabolism pathways (metabolic programs associated with more differentiated, less proliferative hepatocytes). This metabolic dichotomy mirrors prior observations that loss of SDH activity in HCC leads to a switch from aerobic respiration to glycolysis (the Warburg phenotype) and more aggressive tumor growth [24], 43]. In essence, our SMRG signature may reflect aspects of this metabolic reprogramming, thus stratifying tumors by malignant potential. From a clinical standpoint, such a stratification may have potential value in informing post-surgical management strategies. Early-stage HCC patients are generally treated with curative intent (surgery or ablation), but a substantial proportion still experience disease progression after treatment [44], 45]. Current staging systems do not always reliably identify patients with poor postoperative outcomes. Our SMRGs risk score could help identify high-risk individuals who might benefit from closer surveillance or adjunctive therapy after resection. For instance, patients with high SMRG-risk early tumors may be considered for further investigation in the context of clinical trials of adjuvant therapy, while low-risk patients might be comfortably observed. This concept gains support from emerging evidence in HCC, where adjuvant immunotherapy, such as atezolizumab plus bevacizumab, has been evaluated in patients at high risk following curative resection [46]. Although further validation is required (and updated data have nuanced the initial results), it is clear that better risk stratification is needed to select patients for such therapies. Metabolic signatures like ours could be one tool to achieve this, complementing histologic factors such as microvascular invasion in defining “high-risk” early HCC.

We acknowledge several limitations. First, our signatures were derived from retrospective public transcriptomic cohorts and therefore require prospective validation in independent, real-world surveillance populations, ideally including liquid-biopsy settings (e.g., cfDNA methylation, circulating RNA/exosomal RNA) to test clinical feasibility and generalizability. Second, our analyses were based on mRNA expression and did not directly quantify succinate abundance or enzymatic activity due to the limitations of the available transcriptomic datasets; multi-omics validation incorporating metabolomics and functional assays is needed to confirm that the inferred SMRG patterns reflect true succinate accumulation and pathway activation. Third, the TCGA early-stage cohort may introduce selection bias (predominantly resected cases with relatively preserved liver function), and the performance of our signatures in cirrhotic patients with very small lesions (≤2 cm) remains uncertain, warranting evaluation in surveillance-detected nodules and benign mimics. Furthermore, the diagnostic model was constructed based on tumor vs. non-tumor liver tissues, which does not fully represent real-world early detection settings. As a result, the high diagnostic performance observed in this study may be overestimated and should be interpreted as reflecting tissue-level molecular discrimination rather than clinical diagnostic utility. Future studies should incorporate clinically relevant comparator groups and validate the model in prospective surveillance cohorts to enhance real-world applicability. In addition, no explicit cross-platform harmonization or threshold transfer was performed between the TCGA and GEO cohorts which may limit cross-study comparability and introduce platform-dependent bias. Future studies should adopt standardized cross-platform normalization strategies and evaluate fixed, clinically transferable cutoff values to improve generalizability. Besides, due to incomplete staging information in GEO cohorts, strict restriction to early-stage HCC could not be fully applied in external validation datasets, which may introduce clinical heterogeneity and affect comparability with the TCGA cohort. Future validation in well-annotated, uniformly staged cohorts is warranted to enable more rigorous assessment in strictly defined early-stage populations. Moreover, the sequential feature selection strategy applied within the same training cohort may introduce a degree of circularity and increase the risk of overfitting. The lack of internal resampling procedures may further lead to optimistic performance estimates. Therefore, future studies are warranted to adopt more rigorous model development frameworks to improve the robustness of the proposed model. Finally, although we performed exploratory analyses of drug sensitivity and immunotherapy response, these findings were based on predictive models and external cohorts, including the IMvigor210 urothelial carcinoma cohort, which may limit their direct applicability to HCC. Therefore, prospective studies are required to validate the predictive value of the risk model for immune checkpoint blockade or anti-angiogenic therapies. Despite these limitations, our findings provide a proof-of-concept for succinate metabolism–centric signatures as clinically relevant biomarkers in early-stage HCC.

In conclusion, we have shown that succinate metabolism–related gene signatures offer a promising new tool for early-stage HCC. They show potential for identifying early-stage tumors and stratifying patients by overall survival risk, while also providing insights into the tumor’s metabolic and immunologic state. These results encourage further validation in clinical trials and point toward a more integrated approach to HCC management – one that combines traditional clinical factors with molecular profiles reflecting the tumor’s metabolic wiring and immune milieu. Ultimately, such strategies hold the potential to improve early detection and personalize therapy, thereby improving outcomes for patients with HCC.

## Conclusions

Early-stage hepatocellular carcinoma remains difficult to diagnose and risk-stratify with current clinical approaches. Here, succinate metabolism–related gene signatures addressed both needs: an SMRG-based diagnostic panel robustly discriminated early-stage HCC from non-tumor liver tissue, and a succinate-linked prognostic signature stratified patients into distinct risk groups with significantly different outcomes. High-risk tumors exhibited cell-cycle activation and an immunosuppressive microenvironment, consistent with the oncometabolic and immunomodulatory roles of succinate. Collectively, these findings suggest that succinate metabolism–associated transcriptional alterations may be linked to early-stage HCC aggressiveness and highlight the potential utility of integrating SMRG signatures as molecular biomarkers to complement surveillance and refine post-curative prognostic assessment, although their clinical applicability requires further validation. Prospective validation across diverse cohorts, including evaluation in blood-based assays, is warranted.

## Supplementary Material

Supplementary Material

Supplementary Material

Supplementary Material

Supplementary Material

Supplementary Material

Supplementary Material
