# Supplementary material for: Succinate metabolism–related gene signatures enable diagnosis and prognostic stratification in early-stage hepatocellular carcinoma
Source: Open Med (Wars). 2026 Nov 16;21(1):20261465. doi: 10.1515/med-2026-1465 (PMC13766313; doi:10.1515/med-2026-1465)
Supplement: Supplementary file 1 [file j_med-2026-1465_suppl_006.docx]

Supplementary Table S1: Univariate regression analysis results (p<0.001);

Supplementary Table S2: LASSO (glmnet)-selected candidate genes for prognostic model construction (optimal λ by cross-validation);

Supplementary Table S3: Results of multivariate regression analysis;

Supplementary Table S4: Drug susceptibility prediction results of characteristic genes in the CellMiner database.

Supplementary Figure S1: Single-gene GSEA of the four diagnostic biomarkers (CDKN2A, LIFR, NTF3, and PVALB) in early-stage HCC;

Supplementary Figure S2: Clinicopathological subgroup differences in the SMRG-derived risk score in early-stage HCC (age, sex, stage, and T classification);

Supplementary Figure S3: Stratified Kaplan–Meier survival analyses demonstrating robust prognostic discrimination of the SMRG risk signature across clinicopathological subgroups in early-stage HCC.
